# Insulin and glucose play a role in foam cell formation and function

**DOI:** 10.1186/1475-2840-5-13

**Published:** 2006-06-20

**Authors:** Pavel N Shashkin, Nitin Jain, Yury I Miller, Benjamin A Rissing, Yuqing Huo, Susanna R Keller, George E Vandenhoff, Jerry L Nadler, Thomas M McIntyre

**Affiliations:** 1 Cardiovascular Research Center, University of Virginia, 415 Lane Road, Charlottesville, VA 22903, USA; 2 Dept. Cell Biology, Cleveland Clinic, 9500 Euclid Avenue, Cleveland, OH 44195, USA; 3 Pfizer, Inc., Groton, CT 06340, USA; 4 Dept. of Medicine, University of California at San Diego, 9500 Gilman Road, La Jolla, CA 92093, USA; 5 Dept. of Medicine, University of Minnesota, 420 Delaware St SE, Minneapolis, MN 55455, USA; 6 Dept. of Internal Medicine/Division of Endocrinology, University of Virginia, PO Box 801409, Charlottesville, VA 22908, USA

## Abstract

**Background:**

Foam cell formation in diabetic patients often occurs in the presence of high insulin and glucose levels. To test whether hyperinsulinemic hyperglycemic conditions affect foam cell differentiation, we examined gene expression, cytokine production, and Akt phosphorylation in human monocyte-derived macrophages incubated with two types of oxidized low density lipoprotein (LDL), minimally modified LDL (mmLDL) and extensively oxidized LDL (OxLDL).

**Methods and results:**

Using Affymetrix GeneChip^® ^arrays, we found that several genes directly related to insulin signaling were changed. The *insulin receptor *and *glucose-6-phosphate dehydrogenase *were upregulated by mmLDL and OxLDL, whereas *insulin-induced gene 1 *was significantly down-regulated. In hyperinsulinemic hyperglycemic conditions, modified LDL upregulated Akt phosphorylation and expression of the insulin-regulated aminopeptidase. The level of proinflammatory cytokines, IL-lβ, IL-12, and IL-6, and of a 5-lipoxygenase eicosanoid, 5-hydroxyeicosatetraenoic acid (5-HETE), was also increased.

**Conclusion:**

These results suggest that the exposure of macrophages to modified low density lipoproteins in hyperglycemic hyperinsulinemic conditions affects insulin signaling and promotes the release of proinflammatory stimuli, such as cytokines and eicosanoids. These in turn may contribute to the development of insulin resistance.

## Background

Cardiovascular disease (CVD) affects more than 58 million Americans and remains the most common cause of death in the U.S., with atherosclerosis accounting for the majority of these deaths [1,2]. CVD is also the leading cause of mortality in type 2 diabetics [3], and frequently precedes the manifestation of type 2 diabetes [4]. Pathogenic mechanisms involved in early atherosclerosis and the links between CVD, atherosclerosis and diabetes are not well understood.

A critical event in the early stages of atherosclerosis is the focal accumulation of lipid-laden foam cells, largely derived from macrophages, with subsequent fatty streak formation. Foam cell formation, which is believed to be mediated by modified LDLs [5,6], often occurs in the presence of increased concentrations of insulin and glucose. These increased concentrations are characteristic of insulin resistance associated with diabetes, obesity and the metabolic syndrome. Hyperinsulinemia has recently been reported as a risk factor for atherosclerotic diseases such as coronary heart disease [7]. We considered that additive or synergistic effects of modified LDL, insulin and glucose may play a role in foam cell formation and function. Whether or not the processes involved in foam cell formation modulate insulin signaling, glucose tolerance, and onset of type 2 diabetes, is currently unknown.

Macrophages represent a huge reservoir of insulin-sensitive cells. However, they are different from other "classical" insulin-responsive tissues and cells, such as muscle and adipocytes, because they lack the insulin-sensitive glucose transporter GLUT4 [8]. Additionally, insulin receptor substrate 2 (IRS2) evidently plays a more important role than IRS-1 in macrophages [9]. Macrophage infiltration into adipose tissue plays a significant role in obesity-related insulin resistance [10]. Like adipocytes, monocytes/macrophages produce large quantities of biologically active molecules such as tumor necrosis factor alpha (TNFα) and IL-6 [11,12]. In contrast to other cells, the effect of insulin on macrophages has been poorly investigated, with the exception of its involvement in apoptosis [7] and on expression of CD36 [9].

Modified LDL is a collective term for various modifications of native LDL molecules (nLDL). Here we tested whether minimally modified LDL (mmLDL) [13] and fully oxidized LDL (OxLDL), which represent naturally occurring products of LDL with various degrees of oxidation, have effects on insulin signaling in macrophages. The uptake of OxLDL is mediated by a large family of scavenger receptors (rev. in [14]). Since OxLDL induces the expression of some scavenger receptors such as CD36 and SR-A [15], it may provide a positive feedback mechanism that could amplify foam cell formation. mmLDL mainly interacts with the CD14/Toll-like receptor 4 [13]. It is of interest that CD14 antigen can be also upregulated by OxLDL [14,16,17].

In this study we show that modified LDL in the presence of hyperglycemia and hyperinsulinemia promote the production of proinflammatory cytokines and affect insulin signaling in monocyte-derived foam cells. These results suggest that the atherogenic process that is associated with increased LDL uptake by macrophages and foam cell formation may contribute to the progression of insulin resistance and the development of diabetes.

## Materials and methods

### Reagents

OxLDL and mmLDL used in the experiments were prepared from the same native LDL each time as in Miller et al. [18]. Oxidized LDL were analyzed by measuring thiobarbituric reactive substances (TBARS, 30–40 μmol/g protein) and EO6-reactive phospholipid oxidation products. mmLDL contained early lipid peroxidation products, but it did not contain any measurable TBARS or EO6-reactive substances above that of native LDL [13,19]. The mmLDL modification appeared to be very reproducible, and a successful modification was documented in a biological assay in which mmLDL induced spreading of J774 macrophages in cell culture [13,19]. All LDLs were tested for endotoxin level with a LAL kit (BioWittaker, Walkersville, MD). The level of endotoxin contamination was below 2.5 pg/ml. All other chemicals, if not further specified, were from Sigma (St. Louis, MO).

### Donors

Experiments were performed with cells isolated from healthy human subjects who were volunteers recruited by announcements. There were no restrictions regarding sex, age, and ethnicity. All participants signed a consent form approved by the Human Investigation Committees at the University of Virginia, Charlottesville, VA and at Cleveland Clinic, Cleveland, OH. Whole blood (100 ml) was drawn from the antecubital vein of donors. Dextran 500 was added to the blood for sedimentation of red blood cells. After incubation at room temperature, the buffy coat was removed and placed on Histopaque 1.077 (Sigma Diagnostics, Inc., St. Louis, MO). As an alternative to whole blood, we used buffy coats delivered by Virginia Blood Services (Richmond, VA). These buffy coats were used for Affymetrix GeneChip^® ^U133A arrays. Following centrifugation, the mononuclear layer was removed and washed with PBS containing 0.02% EDTA.

### Isolation of monocytes from human blood or buffy coat

Monocytes were isolated using adherence to plastic as in Bey et al. [20]. Briefly, the mononuclear cell pellet was resuspended in 1X H-lyse Buffer (R&D Systems Inc., Minneapolis, MI), and then washed with 1X Wash Buffer. Cells were resuspended in RPMI 1640 +10% FCS (Invitrogen Corporation, Carlsbad, CA) and plated on 60 mm or 100 mm dishes. After one hour incubation at 37°C, the dishes were washed twice with PBS and all non-adherent cells were removed.

### Generation of monocyte-derived macrophages (MDM) and foam cells

For Affymetrix GeneChip^® ^experiment, isolated adherent monocytes were resuspended in Macrophage Serum-Free Medium (MSFM, Gibco, Invitrogen Corporation, Carlsbad, CA) in the presence of 1% media supplement Nutridoma-HU (Roche Molecular Biochemicals, Indianapolis, IN). The concentration of insulin (500 nM) and glucose (17.5 mM) in this medium allowed us to mimic conditions of impaired glucose tolerance or type 2 diabetes (hyperinsulinemia, hyperglycemia). This insulin concentration is greater than in diabetic patients [21]. However, this is a regular insulin concentration for the prolonged cultivation of the cells under serum-free conditions.

For additional experiments, we used an insulin- and glucose-deficient medium (glucose-free RPMI 1640 (Invitrogen Corporation, Carlsbad, CA)+10% dialyzed FCS (HyClone, Logan, UT)) supplemented with varying concentrations of both glucose and insulin. Cells incubated in MSFM and in insulin- and glucose-deficient medium in the presence of 500 nM insulin and 17.5 mM glucose showed similar pattern of gene and protein expression as assessed by RT-PCR and flow cytometry (data not shown). Differentiation of monocytes was induced by macrophage colony-stimulating factor (M-CSF) or platelet factor 4 (PF_4_) (each at concentration 100 ng/ml). We used these stimulators since they have been identified in human atherosclerotic lesions [22,23] and play a pathophysiological role in atherogenesis.

After 6–8 days of incubation with M-CSF or PF_4 _in a CO_2_-incubator (95% O_2_/5% CO_2_), the cell medium was changed for a similar one without stimulator, but with native LDL, OxLDL or mmLDL (100 μg of protein/ml) and/or different concentrations of insulin and glucose. Directly before use, all LDLs were filtered through a 0.45 μ Acrodisc syringe filter to remove aggregates. As a negative control, cultured monocytes/macrophages were exposed to a medium without additives under the same experimental conditions. Cells were further incubated in a CO_2 _incubator for 48 h. Some dishes with differentiated macrophages were also incubated either with monocyte chemoattractant protein-1 (MCP-1 or CCL2) or growth-regulated oncogene alpha (GROα or CXCL1) for 5 h to investigate an acute effect of CC or CXC chemokines. CCL2 and CXCL1/CXCL8 are among the chemokines that have been implicated most strongly in atherogenesis [24]. Data on CCL2 and CXCL1 effects were used only as input data (in combination with other data generated from MDM) with the intent to increase reliability and decrease standard deviations of Hierarchical Clustering and Self Organizing Map analysis. Cell concentration and viability after incubation were assessed using a Guava Personal Cytometer PCA (Guava Technologies, Inc., Hayward, CA).

### RNA isolation and design of Affymetrix GeneChip^® ^U133A array

Total RNA was isolated using RnEasy Mini Kit (Qiagen Inc., Valencia, CA), treated by RNase-free DNase (Qiagen Inc.) to avoid possible DNA contamination and was used for cRNA synthesis. cRNA was used for hybridization to a total of eighteen Affymetrix GeneChip^® ^U133A arrays (nine conditions in duplicates, the conditions are defined in the abscissa of Figure 4 and in Additional figure 1 in Additional file 1). Additional plates were used for confirmation of Affymetrix array data by RT-PCR and for analysis of proteins by Western blot. Aliquots of supernatants were used for detection of 5-HETE and cytokine production by macrophages.

### Real Time RT-PCR

Real time reverse transcriptase-polymerase chain reaction was performed on RNA isolated from MDM and foam cells cultivated under varying concentrations of insulin and glucose. The primers used to analyze mRNA for insulin receptor were: sense-TGCTGGTGTTCATCAGAACAG and antisense-ACAAACACCACCAGGTACATG. Following the reverse transcription step (30 min at 50°C), PCR was performed at 94°C (15 seconds), 60°C (30 seconds), 72°C (30 seconds) with data collection at 79.5°C (15 seconds) for 40 cycles. SYBR Green I (Molecular Probes, Eugene OR) was used as the fluorescent reporter. The specificity of the product was confirmed by analysis of characteristic melting curve and by electrophoresis. Negative controls included RNAse I treatment and omission of the reverse transcription step. Upon treatment with DNAse I, there was no significant change in insulin receptor mRNA expression, indicating an absence of genomic DNA in the samples. Glyceraldehyde-3-phosphate dehydrogenase (GAPDH) was used as an internal standard, and all data were normalized to GAPDH expression. Real Time RT-PCR was performed using an Applied Biosystems ABI Prism 7700 Sequence Detection System.

### Cholesterol and cytokine analysis

Following incubations, MDM cultures were rinsed three times with PBS without Ca^2+ ^and Mg^2+^, scraped into 1 ml of double distilled water and kept frozen at -80°C until chemical analysis was performed. Total and esterified cholesterol was determined enzymatically with the fluorescence method described by Gamble [25]. Macrophage protein content was determined by the Lowry method using BSA as a standard. Cytokine analysis was performed using Cytometric Bead Array kit (BD Pharmingen, San Diego, CA) according to standard manufacturer protocols. High performance liquid chromatography (HPLC) was performed under at least two different conditions for clear identification of 5(s) HETE peak (please see Additional file 1 for details).

### Statistics

Data were compared with either 1-way ANOVA followed by Bonferroni Correction Post Hoc test or Student T test to evaluate 2-tailed levels of significance. Determining Pearson correlation coefficients compared continuous variables. Local Pooled Error (LPE) method [26] was used to determine statistical differences between the changes in the gene expression on the chips.

Two algorithms, Hierarchical Clustering analysis [27] and Self Organizing Map (SOM) [28] were used to identify the clusters of genes that are concomitantly regulated by various LDLs. The GENECLUSTER software [28] produced and displayed SOMs of gene expression data. Genes in biologically interesting clusters identified by these approaches were searched across bioinformatic database GenMAPP.

## Results

### Cholesterol content

**Figure 1:**
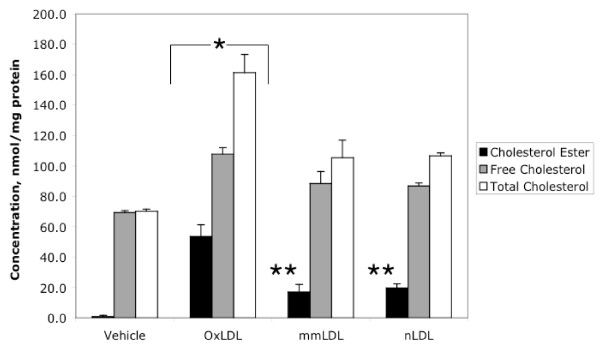
Free, esterified and total cholesterol in the M-CSF-differentiated macrophages cultivated under HIHG conditions. *: p < 0.03 for OxLDL-treated cells vs. Vehicle-treated cells. **: p < 0.05 for mmLDL- and nLDL-treated cells vs. Vehicle-treated cells. Data represent the means ± SEM of triplicates. Black bar – cholesterol ester, grey bar – free cholesterol, open bar – total cholesterol.

We measured cholesterol content as a direct indicator of foam cell formation (Figure 1). As expected [1,29], the largest accumulation of cholesterol was detected in OxLDL-treated cells.

### Analysis of cytokine production by macrophages

**Figure 2:**
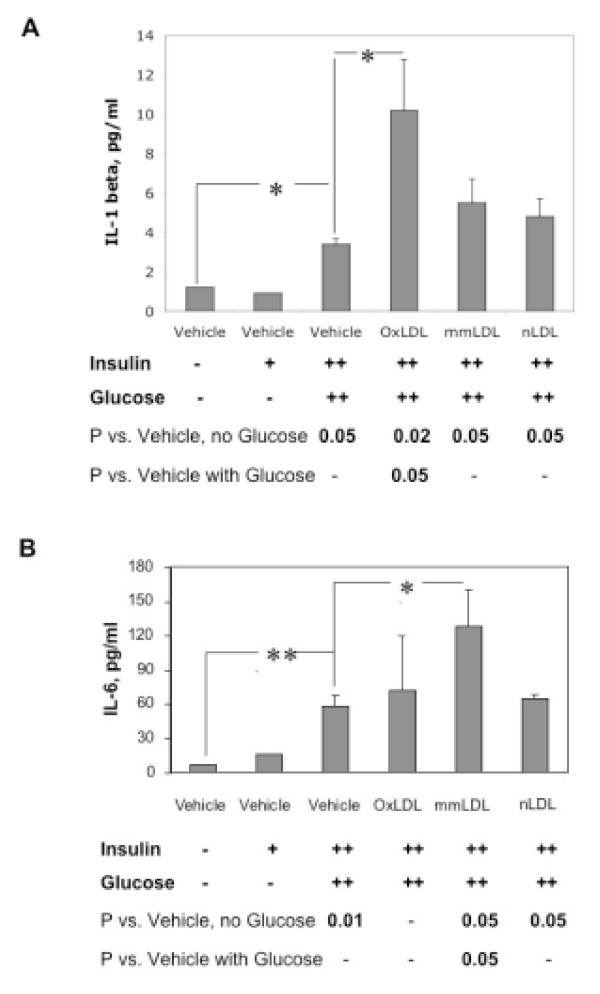
Level of IL-lβ (A) and IL-6 (B) in supernatant after incubation of macrophages with various types of LDL, insulin (+: 10 nM, ++: 500 nM) and glucose (++: 17.5 mM). *: p < 0.05, **:p < 0.01.

Next, we measured inflammatory cytokines in cell supernatants of macrophages incubated with various types of LDL. To identify the direct insulin effect on cytokine production by macrophages, MDM were incubated in insulin-deficient media or with 10 nM insulin (Figure 2, first 2 columns). A moderate dose of insulin (10 nM) did not affect the production of IL-lβ, whereas high insulin (500 nM) in combination with high glucose significantly increased IL-lβ, and the response was further exacerbated by OxLDL (Figure 2A). Similar data was obtained for IL-12 (not shown). IL-6 [30] was also increased under high glucose high insulin conditions, but the effect was further exacerbated specifically by mmLDL (Figure 2B).

### Effect of LDL treatment on gene expression

Next, we examined the effect of LDL treatment on gene expression in macrophages incubated under HIHG conditions using Affymetrix GeneChip^® ^arrays. Affymetrix arrays generated an enormous amount of data that were analyzed by comparing vehicle-treated cells with the cells treated with modified LDL. Among other genes, we found that many genes directly related to insulin signaling were significantly modulated by modified LDL. They include the *insulin receptor, IRS2 *(expressed in monocytes at much higher level than IRS-1 [9] and our unpublished data), the *catalytic subunit δ of phosphatidylinositol 3' kinase *(*PIK3CD*, subunit *δ *is predominantly expressed in leukocytes [31]), *Insulin-Induced gene 1 *and *glucose-6-phosphate dehydrogenase *gene. Both OxLDL and mmLDL significantly upregulated the expression *of glucose-6-phosphate dehydrogenase *(2.3-and 2.0-fold, respectively, p < 0.000001 for both values). OxLDL downregulated the expression of the *Insulin-Induced gene 1 *and the *catalytic subunit δ of phosphatidylinositol 3' kinase *5.4-and 1.9-fold, respectively (p < 0.000001 for both values), but upregulated the expression of the genes encoding the *insulin receptor *and *IRS2 *3.9- and 2.4-fold (p < 0.004 and p < 0.000001), respectively. mmLDL downregulated the expression of the *Insulin-Induced gene 1 *1.4-fold (p < 0.05), and upregulated the expression of the *insulin receptor *gene 1.7-fold (p < 0.05), but had no effect on the *IRS2 *and *the phosphatidylinositol 3' kinase *genes. The changes in expression of these genes by OxLDL and mmLDL are shown in Table 1. The last four genes were significantly correlated across 9 experimental conditions: while *insulin receptor *and *IRS2 *genes were positively correlated, they were negatively related to the genes encoding *PIK3CD *and *Insulin-Induced gene 1 *(Table 2).

**Table 1:** Genes related to the insulin signaling cascade which are specifically modulated by OxLDL in comparison to mmLDL and vehicle in hyperinsulinemic hyperglycemic conditions.

		Mean absolute intensity					
					
Description	Locus Link	Vehicle	mmLDL	OxLDL	Fold change: OxLDL vs. Vehicle	P: OxLDL vs. Vehicle	P: OxLDL vs. mmLDL
*Insulin receptor*	3643	**19.7**	**33.0**	**76.8**	**3.90**	**<0.004**	**<0.009**
*Insulin receptor substrate 2 (IRS2)*	8660	**193.0**	**199.0**	**471.1**	**2.44**	**<0.000001**	**<0.000001**
*Glucose-6-phosphate dehydrogenase*	2539	**1665.8**	**3410.6**	**3866.6**	**2.32**	**<0.000001**	N.S.*
*Insulin induced gene 1 (Insig-1)*	3637	**1485.7**	**1024.8**	**274.6**	**-5.41**	**<0.000001**	**<0.000001**
*Phosphoinositide-3-kinase. Catalytic; delta polypeptide (PIK3CD)*	5293	**1890.4**	**1427.2**	**984.2**	**-1.92**	**<0.000001**	**= 0.001**

*) N.S. – not statistically significant (p > 0.05).

**Table 2:** Correlations between gene expression of insulin signaling cascade-related genes (across 9 experimental conditions performed in duplicates, p < 0.005 for all correlation coefficients).

	*Insulin receptor*	*IRS2*	*Insig-1*	*PIK3CD*
*Insulin receptor*	*-*	0.92	-0.87	-0.93
*IRS2*	0.92	-	-0.94	-0.88
*Insig-1*	-0.87	-0.94	-	0.88
*PIK3CD*	-0.93	-0.88	0.88	-

**Figure 3:**
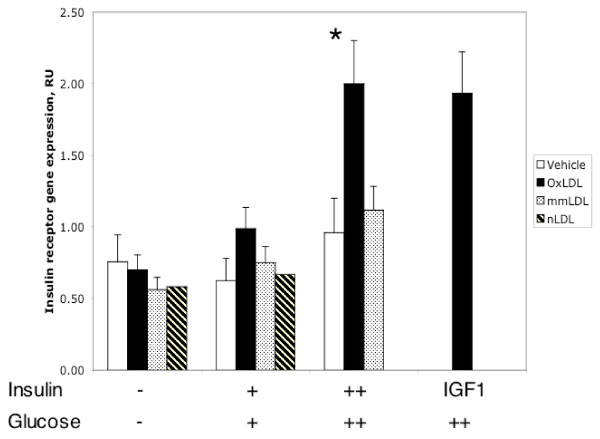
Insulin receptor gene expression as estimated by real-time RT-PCR at various concentrations of insulin (+: 10 nM, ++: 500 nM) and glucose (+: 5.5 mM, ++: 17.5 mM). Only the effect of OxLDL is shown for IGF1. RU, relative units. *: p < 0.05 vs. all other values except for IGF1-treated cells. Means ± SEM of triplicates.

We performed real-time RT-PCR analysis for the *insulin receptor (INS-R) *(Figure 3) and *PIK3CD *(data not shown) to confirm the data of the Affymetrix GeneChip^® ^arrays and to obtain additional data under different experimental conditions, i.e. lower concentrations of insulin and glucose. The results obtained by the Affymetrix GeneChip^® ^arrays (Table 1) were confirmed for OxLDL (Figure 3) and specificity of the change for HIHG conditions was demonstrated. Since insulin at high concentrations may act through the receptors for IGF-1 [32], we evaluated whether IGF-1 has similar effects on the expression of the insulin receptor *(INS-R)*. Indeed IGF-1 in combination with high glucose induced INS-R expression (Figure 3).

**Figure 4:**
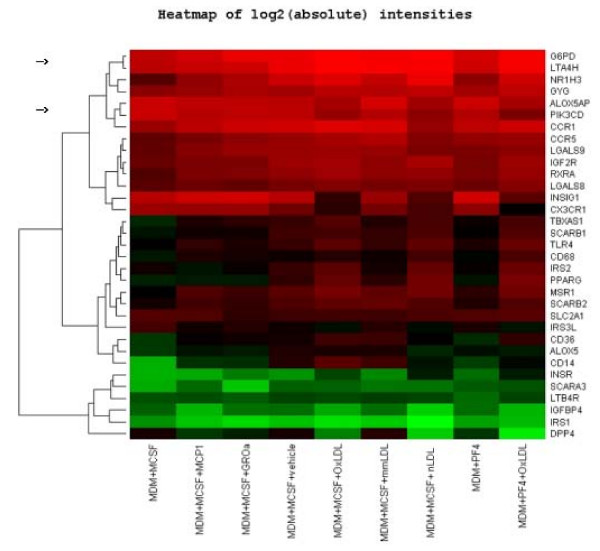
Hierarchical gene clustering of the genes expressed in hyperinsulinemic hyperglycemic conditions (a 18-chip experiment). Green colour corresponds to lowest and red to the highest level of expression of every gene. Log of absolute expression. Arrows indicate clusters to which belong *G6PD*, Glucose-6-phosphate dehydrogenase, and *PIK3CD*, Phosphoinositide-3-kinase (catalytic subunit δ), respectively.

### Identification of the genes regulated concomitantly with the genes of the insulin signaling cascade

To identify the clusters of genes that are concomitantly regulated by various LDLs, Hierarchical Clustering analysis [27] was applied. An example is shown in Figure 4. The genes encoding *Glucose-6-phosphate dehydrogenase (G6PD) *and *Leukotriene A_4 _hydrolase (LTA4H) *belong to the same cluster (two upper rows, Figure 4) as well as the genes encoding *5-lipoxygenase activating protein (ALOX5AP) *and *Phosphoinositide-3-kinase (PIK3CD; *row 5 and 6). The last observation was confirmed using another statistical method, Self Organizing Maps (please see Additional figure 2 in Additional file 1). Arachidonate is a precursor for the biosynthesis of eicosanoids generated by 5-lipoxygenase/ALOX5AP and by other enzymes. Its release from membrane phospholipids is mediated by the activity of phosholipase A_2 _(PLA_2_). We found significant modulation of one of the PLA_2 _isozymes, namely *group IVC cytosolic calcium-independent PLA_2-γ _(PLA2G4C)*. The data on the variations in the genes related to the eicosanoid cascade are presented in Table 3.

**Table 3:** Genes that were modulated by oxidized LDL concomitantly with the genes of the insulin signaling cascade in hyperinsulinemic hyperglycemic conditions.

	Mean absolute intensity						
					
Description	Vehicle	mmLDL	OxLDL	Fold change: OxLDL vs. Vehicle	Fold change: MmLDL vs. Vehicle	P: OxLDL vs. Vehicle	P: MmLDL vs. Vehicle
*Phospholipase A2 (PLA2G4C)*	**455.5**	**266.0**	509.0	1.1	**-1.7**	N.S.*	**<0.0005**
*5-lipoxygenase activating protein (ALOX5AP)*	**1583.3**	**2249.3**	**1160.0**	**-1.4**	**1.4**	**<0.05**	**<0.01**
*Leukotriene A_4 _hydrolase (LTA4H)*	3156.8	3751.3	3743.6	1.2	1.2	N.S.	N.S.

*) N.S. – not statistically significant (p > 0.05).

### Biochemical effects

**Figure 5:**
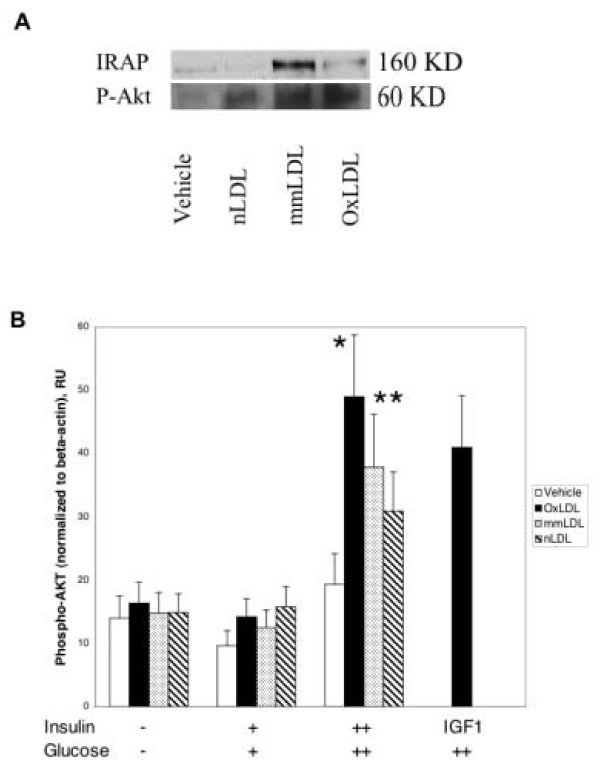
A. Expression of the insulin-regulated aminopeptidase (IRAP, 160 KD) and phosphorylation of PKB/Akt (P-Akt, 60 KD) under hyperinsulinemic hyperglycemic conditions, as analyzed by Western blotting. Equal protein amounts were loaded for each sample. B. Phosphorylation of PKB/Akt as estimated by Western blotting. Results were normalized to β-actin. *,**: p < 0.05 for HIHG conditions vs. other insulin concentrations for OxLDL- and mmLDL-treated cells, respectively. Insulin (+: 10 nM, ++: 500 nM), glucose (+: 5.5 mM, ++: 17.5 mM).

Since we found alterations in the expression of a number of genes belonging to insulin cascade, such as *PIK3CD*, we further explored if the process of foam cell formation under HIHG conditions affects insulin-responsive pathways on protein level. Among several parameters tested, we analyzed the expression of the insulin-regulated aminopeptidase (IRAP) and Akt phosphorylation by Western blot (Figure 5). IRAP represents an effector of insulin action. IRAP expression was stimulated by mmLDL, but slightly inhibited by OxLDL under HIHG conditions. Akt phosphorylation plays a key role in receptor-mediated signaling, including insulin signaling. Both OxLDL and mmLDL significantly increased Akt phosphorylation in macrophages cultured under hyperinsulinemic hyperglycemic conditions (Figure 5A lower and 5B). This effect was probably mediated not only by the insulin receptor, but also by the IGF1-receptor, because the insulin-like growth factor 1 (IGF1) caused a similar response (Figure 5B). Total levels of Akt protein were similar under all conditions examined (not shown).

### Analysis of 5-HETE production by foam cells

**Figure 6:**
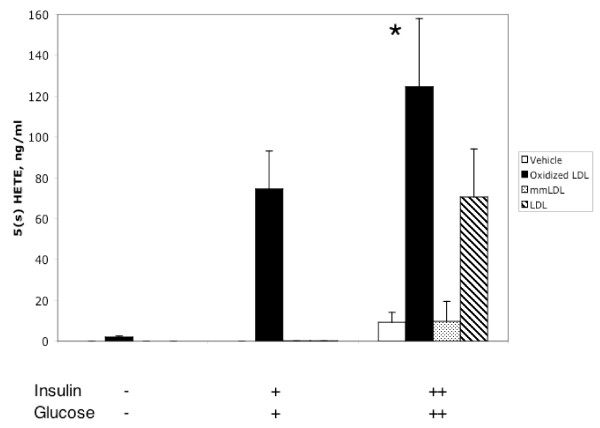
5(s) HETE level in MDM supernatant as analyzed by HPLC at various concentrations of insulin (+: 10 nM, ++: 500 nM) and glucose (+: 5.5 mM, ++: 17.5 mM). *: p < 0.05 vs. all other values. Means ± SEM of triplicates.

Finally, since we found that some genes belonging to the 5-lipoxygenase pathway were regulated concomitantly with the genes of the insulin signaling cascade (see Figure 4 and Additional figure 2 in Additional file 1), we also assessed the levels of the 5-lipoxygenase product 5(s) HETE in the cell supernatants by HPLC. The chromatograms were crowded with many large peaks and chromatography under at least two different conditions had to be performed for clear peak identification (Additional figure 3 in Additional file 1). OxLDL enhanced production of 5(s) HETE in HIHG conditions, as measured by chiral HPLC (Figure 6). OxLDL incubated in the same conditions but without cells did not contain any significant amount of 5(s) HETE (not shown).

## Discussion

Diabetes has been shown to accelerate atherosclerosis. However, it is currently unknown whether proatherogenic processes, such as the formation of oxidized LDL or foam cells, exacerbate the existing state of insulin resistance. In this study, we studied the effect of high doses of insulin and glucose on foam cell formation and cytokine production by macrophages. We found that incubation of MDM with oxidized types of LDL under hyperinsulinemic hyperglycemic conditions led to enhanced secretion of the inflammatory cytokines IL-lβ, IL-6 and IL-12, and the 5-lipoxygenase product 5-HETE. Our data also suggest that foam cell formation in the presence of pathophysiological concentrations of both insulin and glucose *in vivo *leads to the modification of the insulin signaling cascade.

Insulin is a key hormone regulating glucose and lipid metabolism [33,34]. Upon binding insulin, the insulin receptor is activated and subsequently phosphorylates insulin-receptor substrates (IRS) and substrate protein Shc on tyrosine residues. The interaction of tyrosine phosphorylated IRS with the regulatory subunit(s) of phosphatidylinositol 3' kinase p85 activates the catalytic subunit(s) p110. The consequent production of phosphatydylinositol-3-phosphates leads to the phosphorylation and/or activation of downstream targets, including PKB/Akt [33]. mmLDL-induced Akt phosphorylation has been previously described in mouse macrophages [18].

Our findings, specifically the increased expression of *insulin receptor *and *IRS-2 *genes in the presence of OxLDL and HIHG conditions, are consistent with increased insulin signaling. Indeed we found that Akt phosphorylation in human macrophages was upregulated (Figure 5A lower and 5B). It is possible that the effects of high insulin are mediated through the IGF-1 receptor [35], since we could mimic both an increase in *INS-R *expression (Figure 3) and LDL-induced Akt phosphorylation (Figure 5B) using high amounts of IGF-1. However, effects of high IGF-1 may also be mediated by the insulin receptors. Thus, it is still possible that our findings with oxidized LDL are mediated through the insulin and not the IGF-1 receptor.

Insulin affects the activity of genes that have insulin-responsive regions in their promoters. *Glucose-6-phosphate dehydrogenase (G6PD) *is one such example [36]. The recently discovered *Insulin-Induced genes (Insig) *may be responsible for other insulin mediated effects [37,38]. *Insulin-Induced genes 1 and 2 (Insig-1 and Insig-2) *encode proteins of the endoplasmic reticulum that block proteolytic activation of sterol regulatory element-binding proteins (SREBPs). SREBPs are transcription factors that activate the synthesis of cholesterol and fatty acids in the liver and other cells [39]. *Insig *genes are down-regulated by insulin and the fall in Insig expression allows SREBPs to be processed, thereby allowing insulin to stimulate fatty acid synthesis [40]. We detected significant down-regulation of *Insig-1 *gene expression by OxLDL, while mmLDL had a much weaker effect (Table 1). Thus, OxLDL may potentiate the chronic effect of insulin, behaving as an "insulin sensitizer". However, the overall effect of modified LDL on the insulin signaling cascade represents a sum of different effects, including the upregulation of *INS-R *and *IRS2 *genes, and the downregulation of *Insig-1 *and *PIKSCD*. The high correlation present between these four genes (Table 2) indicates that modified LDL may induce a "compensatory shift" in the expression of certain components of the insulin signaling cascade in macrophages cultivated under HIHG conditions.

mmLDL increased the expression of the insulin-regulated aminopeptidase protein (Figure 5A, upper row). IRAP is a member of the family of zinc-dependent membrane aminopeptidases. In fat and muscle cells IRAP localizes in an intracellular compartment under basal conditions and redistributes to the cell surface in response to insulin [41]. Its precise role in insulin action remains unknown [42]. The consequence of increased protein expression of IRAP in insulin signaling in foam cells has to be determined.

The products of 5-lipoxygenase, 5-hydro(pero)xyeicosatetraenoic acid, and the leukotrienes, especially leukotriene B_4_, may also be considered factors mediating the effects of foam cell formation on the insulin signaling cascade. 5-lipoxygenase is upregulated during foam cell formation [43], and its products are believed to play a significant role in inflammation [44,45]. The level of 5-HETE in the cell supernatants was significantly upregulated by OxLDL and positively correlated with the presence of insulin and glucose in cultivation medium (Figure 6).

Insulin resistance and type 2 diabetes are associated with systemic inflammation, possibly through intermediates, such as cytokines and other factors [46,47]. Circulating levels of inflammatory cytokines such as IL-6 and TNFα are increased in type 2 diabetes [48]. Inflammatory cytokines can activate protein kinases that phosphorylate IRS on serine residues, leading to impaired insulin signaling [49]. Despite some controversy regarding the potential role of IL-6 in insulin resistance [50-52], IL-6 has been shown to inhibit insulin signaling and insulin action in isolated hepatocytes [53]. Additionally, IL-6 depletion selectively improves hepatic insulin action in obesity [54]. Moreover, IL-6 leads to insulin resistance *in vivo *when chronically administered to mice at levels that are similar to those found in obese individuals [55]. Hypersecretion of IL-6 and TNFα may exert major stimulatory effects on the synthesis of acute-phase proteins such as PAI-1, which is also related to insulin resistance [56]. Elevated blood levels of IL-lβ and IL-6 increase the risk of type 2 diabetes [47], and lack of IL-lβ decreases the severity of atherosclerosis in ApoE-deficient mice on the C57B1/6 background [57], a model that develops insulin resistance when fed a Western diet [58]. IL-12 is known to favor differentiation of naive T cells along the T-helper (Thl) pathway and play a significant role in atherogenesis [59]. We found OxLDL significantly increased the biosynthesis of IL-lβ and IL-12 by macrophages incubated under HIHG conditions, while mmLDL significantly upregulated IL-6. Increased synthesis of these proinflammatory cytokines in turn can promote insulin resistance in other surrounding cells and tissues.

Dandona et al. noted that increased concentrations of TNFα and IL-6 in type 2 diabetes may modulate insulin action by suppressing insulin signal transduction and this may "shift" the effects of insulin to favor a more proinflammatory state [48]. We hypothesize that oxidized forms of LDL can also "shift" or modulate insulin signaling, at least in macrophages, to a more proinflammatory state. Modified kinds of LDL may bring about effects similar to "selective insulin resistance" [60] or insulin resistance on the level of separate cells/tissues. This could play a role in pathogenesis of "total insulin resistance", thus exacerbating the development of type 2 diabetes. Liang et al. have recently observed that increased CD36 protein expression on mouse macrophages may be connected with defective insulin signaling in these cells as estimated by reduced expression and signaling of insulin receptors [9]. Human macrophages treated with OxLDL *in vitro *indeed show increased surface expression of CD36 [61,62]. Here we report that this change could be associated with an increase in mRNA for insulin receptor and modified insulin signaling. Thus, our results suggest that not exclusively "defective", but rather "modified" or "shifted" macrophage insulin signaling may cause a predisposition to foam cell formation and atherosclerosis in insulin-resistant states.

Our data suggest that the presence or formation of modified/oxidized/aggregated LDL in insulin-resistant patients, who have increased blood insulin and glucose, may exacerbate existing insulin resistance and contribute to the development and progression of type 2 diabetes. Although the treatment of human macrophages with both minimally oxidized LDL or extensively oxidized LDL leads to cholesterol accumulation and foam cell formation *in vitro*, the effects of mmLDL on the insulin signaling cascade in macrophages appear to be less profound or even opposite to those of OxLDL. Such OxLDL-specific effects suggest involvement of the scavenger receptors in a number of downstream events. Further investigations are necessary to evaluate the differential roles of scavenger receptors, including CD36, and CD14/Toll-like receptor 4 in mediating effects of OxLDL and mmLDL, respectively.

## Abbreviations

ALOX5AP = 5-Lipoxygenase activating protein

CVD = Cardiovascular disease

G6PD = Glucose-6-phosphate dehydrogenase

HIHG = Hyperinsulinemic hyperglycemic conditions

HPLC = High performance liquid chromatography

IGF1 = Insulin-like growth factor 1

IL = Interleukin

Insig = Insulin-Induced gene

INS-R = Insulin receptor

IRAP = Insulin-regulated aminopeptidase

IRS2 = Insulin receptor substrate 2

LDL = Low density lipoproteins

LTA4H = Leukotriene A_4 _hydrolase

M-CSF = Macrophage colony-stimulating factor

MDM = Monocyte-derived macrophages

mmLDL = Minimally modified LDL

MSFM = Macrophage Serum-Free Medium

nLDL = Native low density lipoproteins

OxLDL = Oxidized LDL

PF_4 _= Platelet factor 4

PIK3CD = Catalytic subunit δ of phosphatidylinositol 3' kinase

PKB/Akt = Protein kinase B

PLA2 = Phospholipase A_2_

PLA2G4C = Group IVC cytosolic calcium-independent PLA_2-γ_

SOM = Self Organizing Map

SREBP = Sterol regulatory element-binding protein

TNFα = Tumor necrosis factor alpha

5-HETE = 5-Hydroxyeicosatetraenoic acid

## Competing interests

The authors declare that they have no competing interests.

## Authors' contributions

PS conceived of the study, carried out the molecular biological studies and drafted the manuscript. NJ performed the statistical analysis. YM participated in the design of the study, prepared and tested LDL for the study. BR carried out the cytokine assays. YH participated in the design of the study. SK participated in the design of the study and performed protein analysis. GV performed HPLC. JN participated in the design of the study. TM participated in the design and coordination and helped to draft the manuscript. All authors read and approved the final manuscript.

## Supplementary Material

Additional File 1 this file contains additional methods, additional references, and three additional figures (one figure with insert). Click here for file

## References

[B1] Lusis AJ (2000). Atherosclerosis. Nature.

[B2] Streblow DN, Orloff SL, Nelson JA (2001). Do pathogens accelerate atherosclerosis?. J Nutr.

[B3] Candido R, Srivastava P, Cooper ME, Burrell LM (2003). Diabetes mellitus: a cardiovascular disease. Curr Opin Investig Drugs.

[B4] Rett K (1999). The relation between insulin resistance and cardiovascular complications of the insulin resistance syndrome. Diabetes Obes Metab.

[B5] de Villiers WJ, Smart EJ (1999). Macrophage scavenger receptors and foam cell formation. J Leukoc Biol.

[B6] Osterud B, Bjorklid E (2003). Role of monocytes in atherogenesis. Physiol Rev.

[B7] lida KT, Suzuki H, Sone H, Shimano H, Toyoshima H, Yatoh S, Asano T, Okuda Y, Yamada N (2002). Insulin inhibits apoptosis of macrophage cell line, THP-1 cells, via phosphatidylinositol-3-kinase-dependent pathway. Arterioscler Thromb Vasc Biol.

[B8] Daneman D, Zinman B, Elliott ME, Bilan PJ, Klip A (1992). Insulin-stimulated glucose transport in circulating mononuclear cells from nondiabetic and IDDM subjects. Diabetes.

[B9] Liang CP, Han S, Okamoto H, Carnemolla R, Tabas I, Accili D, Tall AR (2004). Increased CD36 protein as a response to defective insulin signaling in macrophages. J Clin Invest.

[B10] Simeoni E, Hoffmann MM, Winkelmann BR, Ruiz J, Fleury S, Boehm BO, Marz W, Vassalli G (2004). Association between the A-2518G polymorphism in the monocyte chemoattractant protein-1 gene and insulin resistance and Type 2 diabetes mellitus. Diabetologia.

[B11] Nishimura F, Iwamoto Y, Mineshiba J, Shimizu A, Soga Y, Murayama Y (2003). Periodontal disease and diabetes mellitus: the role of tumor necrosis factor-alpha in a 2- way relationship. J Periodontol.

[B12] Weisberg SP, McCann D, Desai M, Rosenbaum M, Leibel RL, Ferrante AW (2003). Obesity is associated with macrophage accumulation in adipose tissue. J Clin Invest.

[B13] Miller YI, Viriyakosol S, Binder CJ, Feramisco JR, Kirkland TN, Witztum JL (2003). Minimally modified LDL binds to CD14, induces macrophage spreading via TLR4/MD- 2, and inhibits phagocytosis of apoptotic cells. J Biol Chem.

[B14] Shashkin P, Dragulev B, Ley K (2005). Macrophage differentiation to foam cells. Curr Pharm Design.

[B15] Kita T, Kume N, Minami M, Hayashida K, Murayama T, Sano H, Moriwaki H, Kataoka H, Nishi E, Horiuchi H, Arai H, Yokode M (2001). Role of oxidized LDL in atherosclerosis. Ann NY Acad Sci.

[B16] Nagy L, Tontonoz P, Alvarez JG, Chen H, Evans RM (1998). Oxidized LDL regulates macrophage gene expression through ligand activation of PPARgamma. Cell.

[B17] Han J, Nicholson AC, Zhou X, Feng J, Gotto AM, Hajjar DP (2001). Oxidized low density lipoprotein decreases macrophage expression of scavenger receptor B-I. J Biol Chem.

[B18] Miller YI, Viriyakosol S, Worrall DS, Boullier A, Butler S, Witztum JL (2005). Toll- Like Receptor 4-Dependent and Independent Cytokine Secretion Induced by Minimally Oxidized Low-Density Lipoprotein in Macrophages. Arterioscler Thromb Vasc Biol.

[B19] Miller YI, Worrall DS, Funk CD, Feramisco JR, Witztum JL (2003). Actin Polymerization in Macrophages in Response to Oxidized LDL and Apoptotic Cells: Role of 12/15-Lipoxygenase and Phosphoinositide 3-Kinase. Mol Biol Cell.

[B20] Bey EA, Cathcart MK (2000). In vitro knockout of human p47phox blocks superoxide anion production and LDL oxidation by activated human monocytes. J Lipid Res.

[B21] Niskanen L, Rauramaa R, Miettinen H, Haffher SM, Mercuri M, Uusitupa M (1996). Carotid artery intima-media thickness in elderly patients with NIDDM and in nondiabetic subjects. Stroke.

[B22] Clinton SK, Underwood R, Hayes L, Sherman ML, Kufe DW, Libby P (1992). Macrophage colony-stimulating factor gene expression in vascular cells and in experimental and human atherosclerosis. Am J Pathol.

[B23] Pitsilos S, Hunt J, Mohler ER, Prabhakar AM, Poncz M, Dawicki J, Khalapyan TZ, Wolfe ML, Fairman R, Mitchell M, Carpenter J, Golden MA, Cines DB, Sachais BS (2003). Platelet factor 4 localization in carotid atherosclerotic plaques: correlation with clinical parameters. Thromb Haemost.

[B24] Boisvert WA (2004). Modulation of atherogenesis by chemokines. Trends Cardiovasc Med.

[B25] Gamble W, Vaughan M, Kruth HS, Avigan J (1978). Procedure for determination of free and total cholesterol in micro- or nanogram amounts suitable for studies with cultured cells. J Lipid Res.

[B26] Jain N, Thatte J, Braciale T, Ley K, O'Connell M, Lee JK (2003). Local-pooled-error test for identifying differentially expressed genes with a small number of replicated microarrays. Bioinformatics.

[B27] Eisen MB, Spellman PT, Brown PO, Botstein D (1998). Cluster analysis and display of genome-wide expression patterns. Proc Natl Acad Sci USA.

[B28] Tamayo P, Slonim D, Mesirov J, Zhu Q, Kitareewan S, Dmitrovsky E, Lander ES, Golub TR (1999). Interpreting patterns of gene expression with self-organizing maps: methods and application to hematopoietic differentiation. Proc Natl Acad Sci USA.

[B29] Steinberg D (1997). Low density lipoprotein oxidation and its pathobiological significance. J Biol Chem.

[B30] Huang ZH, Gu D, Mazzone T (2004). Oleic acid modulates the post-translational glycosylation of macrophage ApoE to increase its secretion. J Biol Chem.

[B31] Chantry D, Vojtek A, Kashishian A, Holtzman DA, Wood C, Gray PW, Cooper JA, Hoekstra MF (1997). p110delta, a novel phosphatidylinositol 3-kinase catalytic subunit that associates with p85 and is expressed predominantly in leukocytes. J Biol Chem.

[B32] Buchou T, Gaben AM, Phan-Dinh-Tuy F, Mester J (1991). Insulin/insulin-like growth factor I induce actin transcription in mouse fibroblasts expressing constitutively myc gene. Mol Cell Endocrinol.

[B33] Taha C, Klip A (1999). The insulin signaling pathway. J Membr Biol.

[B34] White MF (2002). IRS proteins and the common path to diabetes. Am J Physiol Endocrinol Metab.

[B35] Entingh-Pearsall A, Kahn CR (2004). Differential roles of the insulin and insulin-like growth factor-I (IGF-I) receptors in response to insulin and IGF-I. J Biol Chem.

[B36] Iritani N (2000). Nutritional and insulin regulation of leptin gene expression. Curr Opin Clin Nutr Metab Care.

[B37] Yang T, Espenshade PJ, Wright ME, Yabe D, Gong Y, Aebersold R, Goldstein JL, Brown MS (2002). Crucial step in cholesterol homeostasis: sterols promote binding of SCAP to INSIG-1, a membrane protein that facilitates retention of SREBPs in ER. Cell.

[B38] Artie AD (2004). Insig: a significant integrator of nutrient and hormonal signals. J Clin Invest.

[B39] McPherson R, Gauthier A (2004). Molecular regulation of SREBP function: the Insig- SCAP connection and isoform-specific modulation of lipid synthesis. Biochem Cell Biol.

[B40] Yabe D, Komuro R, Liang G, Goldstein JL, Brown MS (2003). Liver-specific mRNA for Insig-2 down-regulated by insulin: implications for fatty acid synthesis. Proc Natl Acad Sci USA.

[B41] Keller SR (2003). The insulin-regulated aminopeptidase: a companion and regulator of GLUT4. Front Biosci.

[B42] Keller SR (2004). Role of the insulin-regulated aminopeptidase IRAP in insulin action and diabetes. Biol Pharm Bull.

[B43] Spanbroek R, Grabner R, Lotzer K, Hildner M, Urbach A, Ruhling K, Moos MP, Kaiser B, Cohnert TU, Wahlers T, Zieske A, Plenz G, Robenek H, Salbach P, Kuhn H, Radmark O, Samuelsson B, Habenicht AJ (2003). Expanding expression of the 5- lipoxygenase pathway within the arterial wall during human atherogenesis. Proc Natl Acad Sci USA.

[B44] Radmark O (2003). 5-lipoxygenase-derived leukotrienes: mediators also of atherosclerotic inflammation. Arterioscler Thromb Vase Biol.

[B45] Spanbroek R, Habenicht AJ (2003). The potential role of antileukotriene drugs in atherosclerosis. Drug News Perspect.

[B46] Haffner SM (2003). Insulin resistance, inflammation, and the prediabetic state. Am J Cardiol.

[B47] Spranger J, Kroke A, Mohlig M, Hoffmann K, Bergmann MM, Ristow M, Boeing H, Pfeiffer AF (2003). Inflammatory cytokines and the risk to develop type 2 diabetes: results of the prospective population-based European Prospective Investigation into Cancer and Nutrition (EPIC)-Potsdam Study. Diabetes.

[B48] Dandona P, Aljada A, Bandyopadhyay A (2004). Inflammation: the link between insulin resistance, obesity and diabetes. Trends Immunol.

[B49] Saltiel AR, Pessin JE (2002). Insulin signaling pathways in time and space. Trends Cell Biol.

[B50] Pedersen BK, Steensberg A, Fischer C, Keller C, Keller P, Plomgaard P, Febbraio M, Saltin B (2003). Searching for the exercise factor: is IL-6 a candidate?. J Muscle Res Cell Motil.

[B51] Wallenius V, Wallenius K, Ahren B, Rudling M, Carlsten H, Dickson SL, Ohlsson C, Jansson JO (2002). Interleukin-6-deficient mice develop mature-onset obesity. Nat Med.

[B52] Di Gregorio GB, Hensley L, Lu T, Ranganathan G, Kern PA (2004). Lipid and carbohydrate metabolism in mice with a targeted mutation in the IL-6 gene: absence of development of age-related obesity. Am J Physiol Endocrinol Metab.

[B53] Senn JJ, Klover PJ, Nowak IA, Mooney RA (2002). Interleukin-6 induces cellular insulin resistance in hepatocytes. Diabetes.

[B54] Klover PJ, Clementi AH, Mooney RA (2005). Interleukin-6 depletion selectively improves hepatic insulin action in obesity. Endocrinology.

[B55] Klover PJ, Zimmers TA, Koniaris LG, Mooney RA (2003). Chronic exposure to interleukin-6 causes hepatic insulin resistance in mice. Diabetes.

[B56] Juhan-Vague I, Alessi MC, Mavri A, Morange PE (2003). Plasminogen activator inhibitor-1, inflammation, obesity, insulin resistance and vascular risk. J Thromb Haemost.

[B57] Kirii H, Niwa T, Yamada Y, Wada H, Saito K, Iwakura Y, Asano M, Moriwaki H, Seishima M (2003). Lack of interleukin-lbeta decreases the severity of atherosclerosis in ApoE-deficient mice. Arterioscler Thromb Vasc Biol.

[B58] Phillips JW, Barringhaus KG, Sanders JM, Yang Z, Chen M, Hesselbacher S, Czarnik AC, Ley K, Nadler J, Sarembock IJ (2003). Rosiglitazone reduces the accelerated neointima formation after arterial injury in a mouse injury model of type 2 diabetes. Circulation.

[B59] Davenport P, Tipping PG (2003). The role of interleukin-4 and interleukin-12 in the progression of atherosclerosis in apolipoprotein E-deficient mice. Am J Pathol.

[B60] Montagnani M, Golovchenko I, Kim I, Koh GY, Goalstone ML, Mundhekar AN, Johansen M, Kucik DF, Quon MJ, Draznin B (2002). Inhibition of phosphatidylinositol 3- kinase enhances mitogenic actions of insulin in endothelial cells. J Biol Chem.

[B61] Han J, Hajjar DP, Febbraio M, Nicholson AC (1997). Native and modified low density lipoproteins increase the functional expression of the macrophage class B scavenger receptor, CD36. J Biol Chem.

[B62] Tsukamoto K, Kinoshita M, Kojima K, Mikuni Y, Kudo M, Mori M, Fujita M, Horie E, Shimazu N, Teramoto T (2002). Synergically increased expression of CD36, CLA-1 and CD68, but not of SR-A and LOX-1, with the progression to foam cells from macrophages. J Atheroscler Thromb.

