# Supplementary material for: Insulin and glucose play a role in foam cell formation and function
Source: Cardiovasc Diabetol. 2006 Jun 20;5:13. doi: 10.1186/1475-2840-5-13 (PMC1550220; doi:10.1186/1475-2840-5-13)

# SUPPLEMENTARY DATA

*Design of Affymetrix GeneChip® U133A array* - Cells for Affymetrix GeneChip® array experiments were cultivated in hyperinsulinemic hyperglycemic (HIHG) conditions as specified above. Conditions used for analysis are defined in Additional figure I.

*Self Organizing Map (SOM)* was used to identify the clusters of genes that are concomitantly regulated by various LDLs. An example is presented in Additional figure II.

*High performance liquid chromatography (HPLC) with fluorimetric detection* - Free fatty acids were fluorescinated followed by reverse-phase HPLC [1]. The fluorescent derivatives of the free fatty acids were formed by treatment of the evaporated ethyl acetate fractions with catalyst (N,N-diisopropylethylamine) and dye (2-(2,3-naphthalimino) ethyltrifluoromethanesulfonate). The reaction mixtures were dried with nitrogen, redissolved in 0.4 ml acetonitrile, vortexed, then diluted with 0.6 ml water and applied to a Sep Pak column to remove the reactants. The columns were washed with 50% acetonitrile, and the fatty acid derivatives were eluted with 0.6 ml of ethyl acetate. The fatty acid derivatives were dried with nitrogen, redissolved in 100 μl of methanol, and then loaded into an autosampler glass microinsert for HPLC analysis.

HPLC separation and analysis by fluorescence detection was accomplished by loading the samples onto a C18 Waters symmetry column (Milford, MA), and eluting isocratically for 100 minutes at 61% solvent B = 50% methanol/tetrahydrofuran + 0.1% acetic acid and 39% A = 0.1% acetic acid in acetonitrile. 5-HETE peak was detected fluorimetrically with the excitation wavelength set at 259 nm and emission wavelength at 394 nm. For further separation of 5(s) and non-enzymatic 5(r) HETE isomers, a narrow band of fractions (about 5x1 min fractions at a

retention time of 75 min) centered on the 5-HETE peak position was collected. After drying the pooled peak fractions in a Speedvac and reconstituting in methanol, the peak fraction pool was reinjected onto a chiral phase column (Chiralpak AD, 0.46x25cm - Chiral Technologies Inc., West Chester, PA) and eluted with hexane + 15% ethanol + 3.5% methanol at 1.5 ml/min. The 5(s) and 5(r) HETE derivatives were cleanly separated.

Representative reverse phase chromatogram is presented in Additional figure III with the region around the 5-HETE peak analyzed on the chiral column in insert.

## *Additional reference*

[1] Reilly, K. B., Srinivasan, S., Hatley, M. E., Patricia, M.K., Lannigan, J., Bolick, D.T., Vandenhoff, G., Pei, H., Natarajan, R., Nadler, J.L., Hedrick, C.C.: **12/15-Lipoxygenase activity mediates inflammatory monocyte/endothelial interactions and atherosclerosis in vivo**. *J Biol Chem* 2004, **279**:9440-50.

## *Additional figure legends*

Additional figure I.

Experimental conditions used for Affymetrix GeneChip® U133A array.

Additional figure II.

5x6 Self Organizing Map for 9 experimental conditions. Every cluster (totally 30 clusters) represents a set of genes which are similarly regulated across 9 conditions. N, amount of genes in the cluster. *INSR*, insulin receptor, *IRS2*, Insulin receptor substrate 2, *PLA2G4C*, phospholipase A2, *PIK3CD*, phosphatidyl inositol 3' kinase, *INSIG1*, Insulin Induced gene 1, *ALOX5AP*, 5-lipoxygenase activating protein.

Additional figure III. High performance liquid chromatography (HPLC) of 5-HETE in macrophage supernatant. Representative reverse phase chromatogram of macrophages treated with OxLDL at HIHG conditions. Region around the 5-HETE peak (indicated by the brackets) was collected, dried, reconstituted and run on the chiral column. The result of the analysis is shown in *insert*: Representative chiral HPLC of the collected fraction. 8-HETE served as internal standard.

## Additional figure I

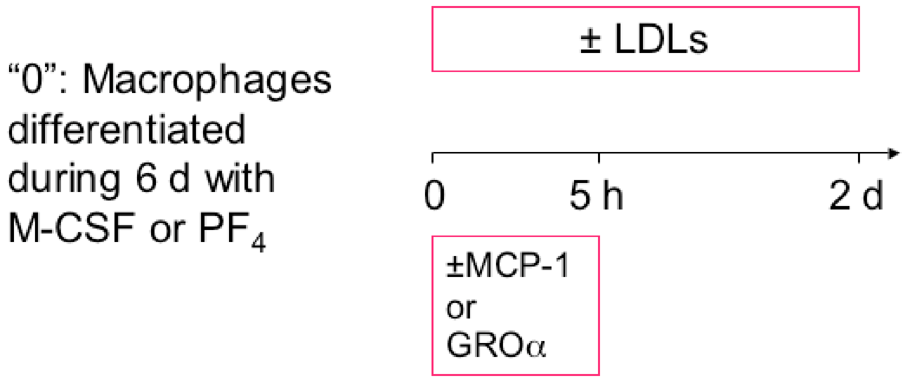

# Additional figure II

**5 x 6 SOM of 9 experimental conditions**

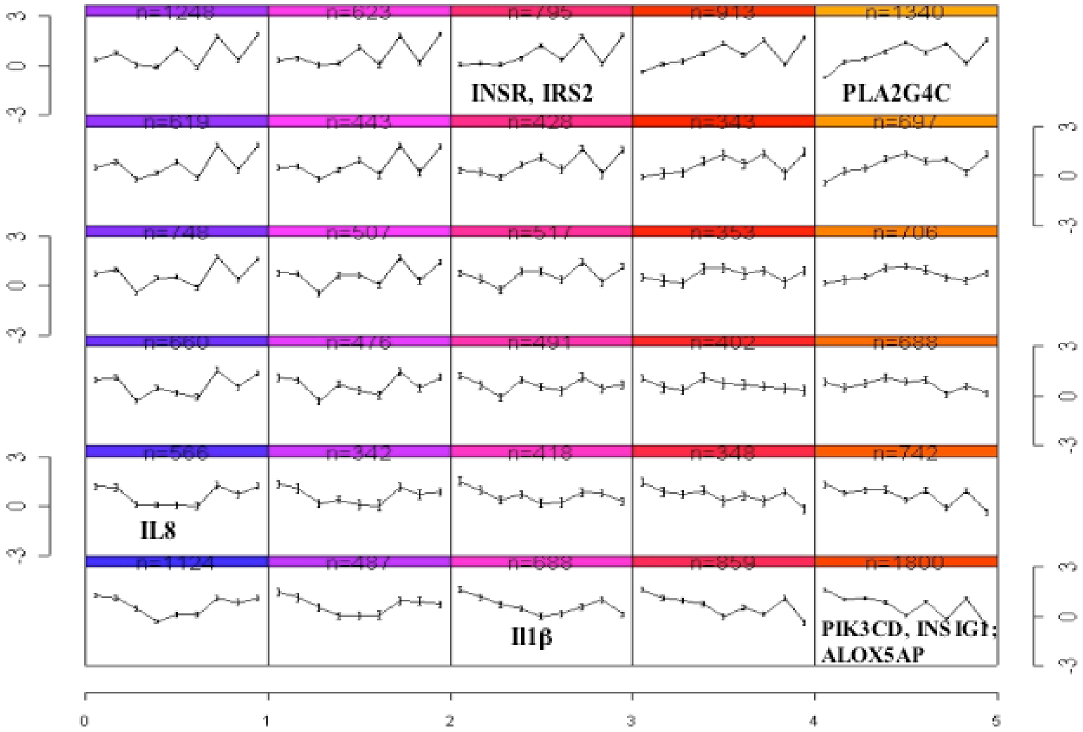

# Additional figure III

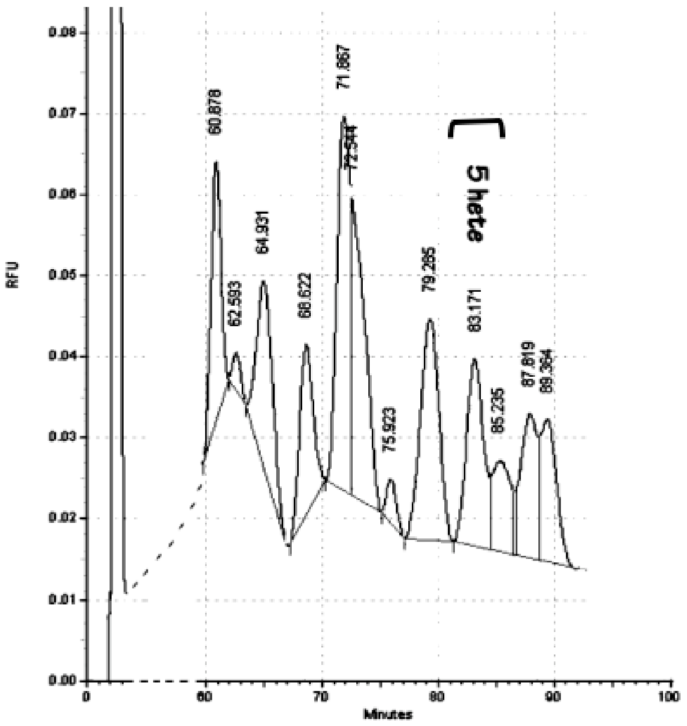


*Insert*

# Insert in Additional figure III

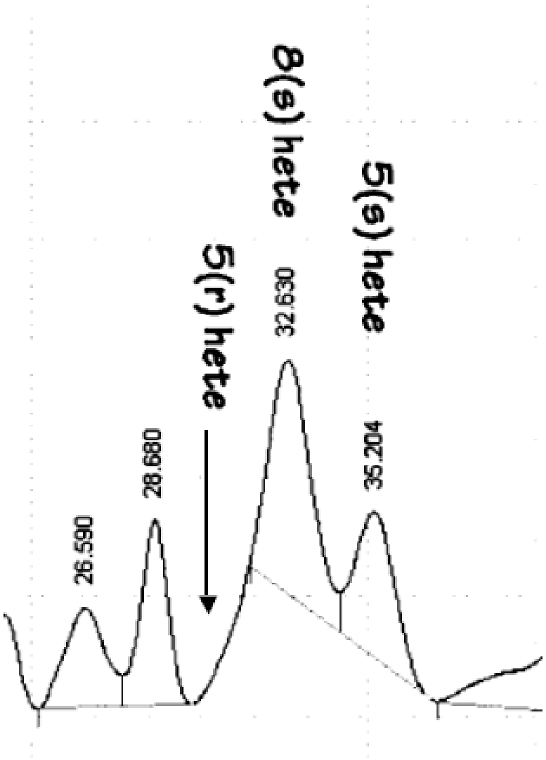

Supplement: Additional File 1 — this file contains additional methods, additional references, and three additional figures (one figure with insert). [file 1475-2840-5-13-S1.pdf]
